# In Search for the Membrane Regulators of Archaea

**DOI:** 10.3390/ijms20184434

**Published:** 2019-09-09

**Authors:** Marta Salvador-Castell, Maxime Tourte, Philippe M. Oger

**Affiliations:** 1 Université de Lyon, CNRS, UMR 5240, F-69621 Villeurbanne, France (M.S.-C.) (M.T.); 2 Université de Lyon, INSA de Lyon, UMR 5240, F-69621 Villeurbanne, France

**Keywords:** Archaea, membrane organization, membrane modulators, polyterpenes, carotenoids, polyprenols, quinones, polyisoprenoids, adaptation

## Abstract

Membrane regulators such as sterols and hopanoids play a major role in the physiological and physicochemical adaptation of the different plasmic membranes in Eukarya and Bacteria. They are key to the functionalization and the spatialization of the membrane, and therefore indispensable for the cell cycle. No archaeon has been found to be able to synthesize sterols or hopanoids to date. They also lack homologs of the genes responsible for the synthesis of these membrane regulators. Due to their divergent membrane lipid composition, the question whether archaea require membrane regulators, and if so, what is their nature, remains open. In this review, we review evidence for the existence of membrane regulators in Archaea, and propose tentative location and biological functions. It is likely that no membrane regulator is shared by all archaea, but that they may use different polyterpenes, such as carotenoids, polyprenols, quinones and apolar polyisoprenoids, in response to specific stressors or physiological needs.

## 1. Introduction

In 1972, Singer and Nicolson reconciled the numerous observations about cell membranes to construct the now well-established fluid mosaic model [1]. Since then, the ultrastructure of cell membranes has further evolved to accommodate the lipid phases, i.e., gel or liquid crystalline, lipid phase partition, membrane curvature and the presence of lipid membrane regulators, which are currently gaining much attention in membrane structuration, function and regulation [2,3,4]. Lipid membrane regulators allow to expand the functional state of the lipid membrane to broader environmental conditions. The best studied membrane regulator is cholesterol, a sterol derivative present in animal cell membranes [3,5,6,7,8,9,10]. Although Bacteria do not synthesize sterols, their hopanoids have been accepted as “sterol surrogates” [11,12]. Sterols or hopanoids are absent in Archaea, and whether Archaea have membrane regulators remains a hotly debated question. The current review sums up the data available on putative archaeal membrane regulators and poses the groundwork for their identification in Archaea.

## 2. Structure of Bacterial and Eukaryal Membrane Regulators

**Figure 1:**
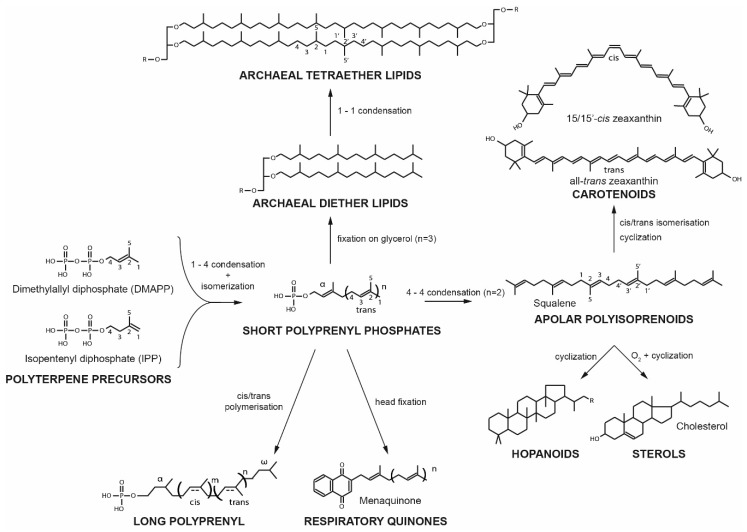
Most representative polyterpenes and their biosynthetic link. Carbon nomenclature and isoprene conformations are indicated as mentioned hereafter.

Sterols are the most well-known lipid membrane regulators. The term sterols covers a variety of compounds synthesized from 2,3-epoxide-squalene and consisting of an aliphatic chain with 7–10 carbons and four flat fused rings, the outermost one exhibiting an sn-3 hydroxyl group [6]. The three major kingdoms of the Eukarya, e.g., mammals, fungi and plants, synthesize different types of sterols, cholesterol, ergosterol and sitosterols and stigmasterols respectively. Hopanoids are pentacyclic triterpenoids synthesized from squalene. Such term regroups C30 derivatives such as diploptene, a hydrophobic molecule, and C35 molecules such as bacteriohopane, and their derivatives. [13]. In hydrophilic hopanoids, hydroxyl groups are bound to the branched aliphatic chain, which therefore results in an “inverted” polarity in comparison to sterols. Sterols and hopanoids belong to a much larger group of natural compounds called polyterpenes, i.e., hydrocarbon oligomers resulting from successive condensations of isoprene precursors, namely, isopentenyl pyrophosphate (IPP) and dimethyallyl diphosphate (DMAP) (Figure 1). Polyterpenes represent one of the largest class of naturally occurring compounds and are widely distributed in Eukaryotes, Bacteria as well as Archaea. Although all polyterpenes of the three domains of life originate from IPP and DMAP, these precursors are synthesized via two independent, non-homologous pathways: the methylerythritol 4-phosphate pathway in Bacteria and the mevalonate pathway in Eukaryotes and Archaea [14,15].

## 3. Mechanisms of Membrane Regulation in Eukarya and Bacteria

The impact of sterols, and particularly of cholesterol, on lipid membranes has been largely studied. Sterols are oriented perpendicular to the membrane surface with the hydroxyl facing the phospholipid ester carbonyl and stabilize the functional phospholipid liquid-crystalline phase, i.e., decrease gel-to-liquid lipid phase transition temperature (T_m_). Sterols modulate membrane parameters by tightening and reducing the average tilt of phospholipid acyl chains [16], therefore decreasing acyl chains’ motion [17] while increasing the viscosity and the order of lipid membranes [18]. They also limit lipid membrane passive permeability to ions and small molecules [17]. Last, cholesterol is the essential component of the thicker liquid-ordered phase present in eukaryotic cell membranes, previously called “lipid raft”, which is essential for membrane functional differentiation. This cholesterol-induced lipid phase leads to a discontinuity of the membrane boundary and, therefore, to a line-tension between both phases [19]. This tension may facilitate cell membrane bending and, consequently, cell processes as fusion and fission, which are essential for numerous physiological mechanisms including cell division, cell compartmentalization or vesicle formation.

The hydrophobic hopanoid, diploptene, is placed in the midplane of the lipid bilayer with an average tilt angle of about 51° to the membrane plane, whereas bacteriohopanetetrol presents an orientation similar to that of cholesterol with an average tilt angle of 14° [20]. Hopanoids, although with different efficiencies, can induce order and decrease fluidity and permeability of model membranes, even though they do not show a significant effect on bilayer elasticity [12,13,20,21]. Similarly to cholesterol, hopanoids can induce the formation of more ordered phases at physiological concentrations [20]. Whether they are involved in membrane domain formation in Bacteria remains to be demonstrated.

## 4. Candidate Surrogates for Sterol and Hopanoid Membrane Regulators in Archaea

As Archaea apparently lack both sterols and hopanoids and considering the physiological importance of membrane regulators in bacterial and eukaryal membranes, we can suppose the existence of membranes regulators in Archaea as well. However, one has to keep in mind that archaeal and bacterial/eukaryal lipids strongly differ in their structure. The former ones being based on ether-linked isoprenoid chains, while the later are ester-linked fatty acids. Regardless, the numerous functions of sterols/hopanoids in the membranes, and especially their importance in maintaining membrane functionality under stress conditions, are coherent with the way of life of Archaea, which generally thrive in the most extreme environments. Since Archaea are supposed to be one of the most ancient phylum on Earth and that isoprenoid lipid synthesis can be traced back to the last universal common ancestor [22], we hypothesize that lipid regulators in Archaea should also originate from the isoprenoid synthesis pathway, be widely distributed in Archaea and impact cell membrane properties. The next sections present an exhaustive collation of the data on the four types of polyterpenes that have been found in Archaea, i.e., carotenoids, polyprenols, quinones and apolar polyisoprenoids.

## 5. Carotenoids

Carotenoids comprise a large group of natural polyterpene pigments synthesized in the three domains of life [23]. They present a characteristic end-group on each side of a polyprenyl chain that usually contains eight or ten isoprene units. To date, about 1200 natural carotenoids have been identified [24] and are classified in two subclasses as a function of the polarity of their end-groups: apolar and polar end-groups for Carotenes and Xantophylls, respectively. Carotenoids may be further divided into two subcategories according to the conformation of their polyprenyl chain. Trans-carotenoids, have only trans-unsaturations, a linear structure and present their functional groups on different sides of the carbon chain. Cis-carotenoids harbor a cis-unsaturation, which induces a kink in the chain and exposes their functional groups on the same side of the carbon chain (Figure 1).

### 5.1. Distribution in Archaea

**Figure 2:**
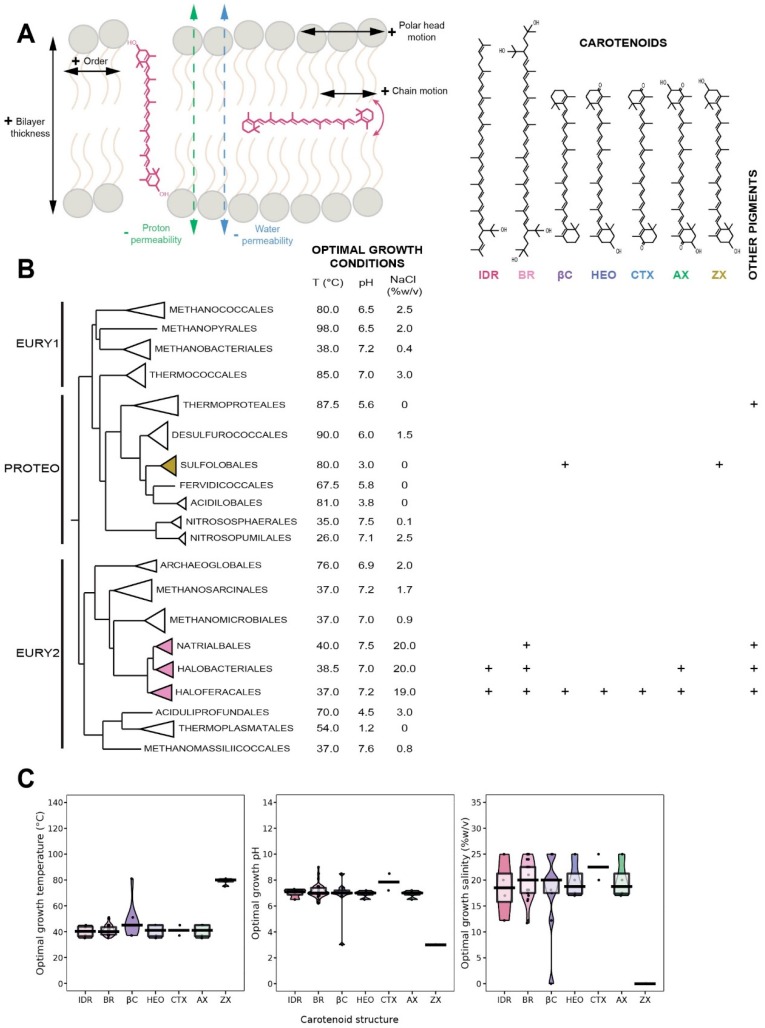
Physicochemistry, distribution and adaptive response of carotenoids in Archaea. (**A**), Position and impacts of carotenoids on membrane physicochemical properties. (**B**), Structures and distribution of carotenoids within the Archaea domain. The tree topology has been adapted from [34] (**C**), Pirateplot of the optimal growth conditions of the organisms in which the different types of carotenoids were detected. Colors indicate the chain lengths as in B. Abbreviations: IDR, isopentenyldehydrorhodopin; BR, bacterioruberin; BC, β-carotene; HEO, 3-hydroechinenone; CTX, canthaxanthin; AX, astaxanthin; ZX, zeaxanthin. EURY1, Euryarchaeota cluster I; PROTEO, Proteoarchaeota; EURY2, Euryarchaeota cluster II.

Very little is known about the distribution of carotenoids in Archaea, as only the Natrialbales, Halobacteriales and Haloferacales orders, as well as few species of the Sulfolobales order were investigated so far (Figure 2A). A diversity of xanthophylls, e.g., zeaxanthin, astaxanthin, canthaxanthin, 3-hydroxyechinenone and bacterioruberin, together with their precursor, isopentenyldehydrorhodopin, were identified in these archaeal orders (Figure 2A) [25,26,27,28,29,30]. In contrast to plants or bacteria which synthesize a large diversity of carotenes, β-carotene is the only carotene identified in Archaea [26,31,32]. Carotenoids may represent a significant fraction of lipids, such as 0.2% (*w/w*) in *Haloferax alexandrinus*, an extreme halophile, most of them being bacterioruberin and canthaxanthin with small quantities of 3-hydroxyechinenone and β-carotene [26]. Similarly, significant quantities of bacterioruberin were found in *Haloferax japonica,* another extremely halophilic archaea [33]. In contrast, only zeaxanthin was identified in the thermoacidophilic archaeon *Sulfolobus shibatae* [25].

### 5.2. Biological Function of Carotenoids

There is only scarce specific data on the biological function of carotenoids in Archaea. However, it is well-established that carotenoids act as antioxidants and protect cell membrane against the oxidative effect of free radicals via direct quenching in Bacteria and Eukarya [35]. The antioxidant effect of carotenoids has been established in vitro [35,36,37] and in vivo [38]. Early studies have shown that the scavenging of radical cations is higher for apolar carotenoids [39], and that the number of unsaturations, the type of end-groups or the membrane lipid composition define their antioxidant properties [40,41]. Carotenoids also play a role in the modulation of the physicochemical properties of membranes [42]. For example, bacterioruberin is an essential part of specific transmembrane proteins [43,44] and controls membrane organization through its high impact on membrane physics and dynamics [45]. Therefore, it is reasonable to assume similar antioxidant or membrane regulator functions in Archaea, especially in the view that many archaea are extremophiles. However, one should not forget that the function of carotenoids is affected by its lipid environment, and thus may significantly differ between Archaea and Bacteria/Eukarya.

### 5.3. Insertion of Carotenoids in the Membrane

The insertion of carotenoids in the membrane depends on their polarity. Apolar carotenoids insert within the hydrophobic part of the lipid membrane. β-carotene, which is the only one found in Archaea, is placed in a bacterial bilayer at 55° from the axis normal to the plane of the membrane [42], although it retains a considerable mobility [46]. In contrast, polar carotenoids, which possess two polar regions placed at each side of the isoprenoid chain, are oriented parallel to the fatty acid chains with their polar end groups anchored in the headgroup regions on both sides of the membrane, therefore physically bridging the two lipid leaflets of the bacterial bilayer. Due to the similar backbone of carotenoids and archaeal lipids, it is probable that both polar and apolar carotenoids may be inserted alongside the isoprenoid chains of the lipids and that their mobility may differ largely from that found in Bacteria. The polar and apolar carotenoids found in Archaea are all based on a β-carotene structure, which length has been estimated at 32 Å and 38 Å for astaxanthin and bacterioruberin, respectively [4,5,21]. Molecular dynamics simulations have found a thickness of 39 Å for the archaeal tetraether monolayer [47]. A similar thickness, 38 Å, is measured for the archaeal bilayer (Salvador Castell/Oger, unpublished results), which means that bacterioruberin may correctly connect both leaflets of the phospholipid bilayer in specific regions of the cell membrane and easily interact with transmembrane proteins. It is still unclear how the known short archaeal polar carotenoids, i.e., hydroechinenone, canthaxanthin, astaxanthin, zeaxanthin, (Figure 2B) would insert in the monolayer of the Sulfolobales or in the bilayer membrane of halophiles.

### 5.4. Carotenoids as Putative Membrane Regulators in Archaea

In Bacteria, carotenoids exhibit numerous similarities with membrane regulators. Furthermore, 10 mol% of polar carotenoids has a similar impact on the structure and dynamic properties of membranes as 15–20 mol% of cholesterol: an increase in order which impacts the rigidity of lipid membranes [48], and decreases membrane fluidity [49] and a decrease in alkyl chain motion (liquid-ordered phase) [36,48,50]. Moreover, carotenoids decrease water [46], small molecules [51] and proton permeability [52] and penetration of oxygen [53], decrease T_m_ by about 1.5–2.5 °C [41] and reduce lipid cooperativity [41] (Figure 2A). Similarly to cholesterol, unsaturations on the lipid hydrocarbon chains decrease the physical impact of xanthophylls [54]. Xanthophylls disturb the membrane polar region [55] and promote the adhesion [56], aggregation and fusion of liposomes [57], which may indicate a change on the intrinsic membrane curvature [58]. Carotenoids display the physicochemical impact on membranes as true membrane regulators, and thus could play this role as well in archaeal membranes, which would constitute a fast and efficient adaptation mechanism to changing external conditions. Although, due to the limited data available, no clear adaptive correlation can be drawn today (Figure 2C), several observations support this view. For example, carotenoids are powerful scavengers of free radicals in halophilic archaea. Indeed, the absence of bacterioruberin in *Halobacterium salinarum* increases the effect of DNA-damaging agents such as UV and ionizing radiations, hydrogen peroxide and mitomycin-C [28,59]. The production of carotenoids is also dependent on growth conditions [60], such as sub-optimal [61,62] or supra-optimal salinity [63,64], illumination and oxygenation [65]. Thus, carotenoids may help prevent cell lysis under non-optimal growth conditions by increasing the stability of cell membranes. Unfortunately, due to the limited data available, no adaptive correlation can be drawn (Figure 2C).

## 6. Polyprenols

Polyprenols are a family of diverse membrane-bound linear polyisoprenoids found in the three domains of life. They have various biological functions, such as biosynthesis of higher terpenes, protein prenylation and glycosylation as well as protection of lipids against peroxidation [66]. Polyprenols have polyisoprenoid chain lengths ranging from 2 up to 100 isoprene units, the eukaryotic polyprenyl alcohols being generally longer (C90–100) than their bacterial and archaeal homologs (C55). Polyprenols have a restricted type of polar headgroups, e.g., alcohol, phosphate or diphosphate. The polyisoprenoid chains are either all-*trans*, such as in the ones involved in terpene synthesis, or of the *cis* type, as is the case for the majority of membrane-bound polyprenols [67]. In polyprenols, the isoprene closest to the polar head is referred as the alpha-unit and the omega-unit is the farthest. Despite their large structural diversity, polyprenols are sorted in only two classes: (1) polyprenols, in which the alpha-unit is unsaturated and (2) dolichols, where the alpha-unit is saturated [68]. Such alpha saturation only has minor impacts on molecular properties as polyprenol and dolichol derivatives behave and locate similarly within membrane bilayers [69]. However, polyprenols are assumed to belong to the dolichol type in Eukarya and Archaea and of the polyprenol type in Bacteria [68].

### 6.1. Distribution in Archaea

**Figure 3:**
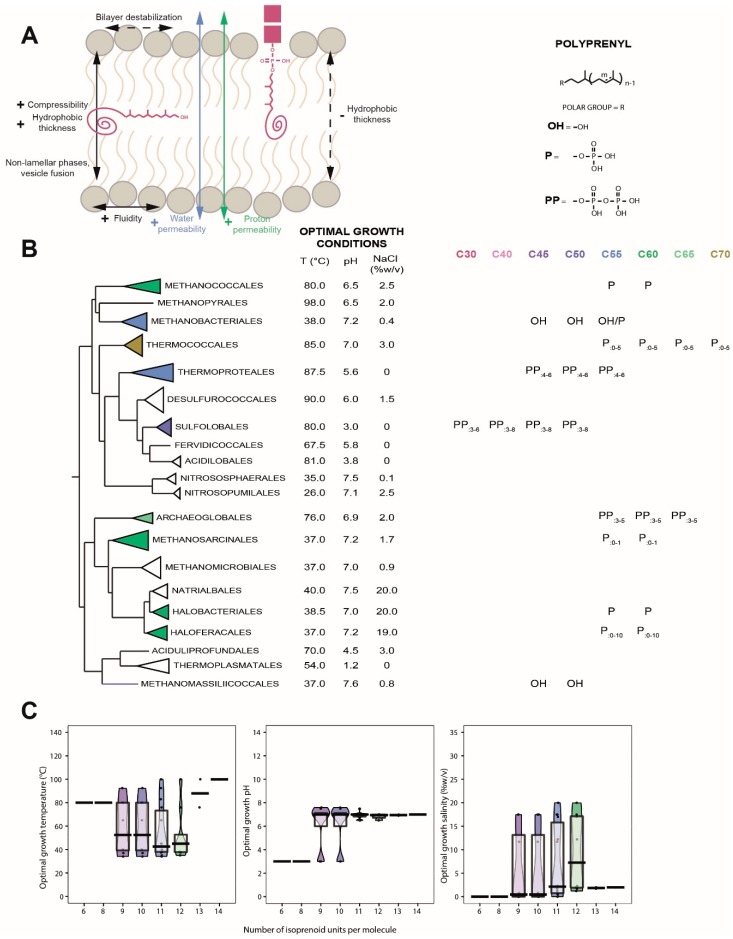
Physicochemistry, distribution and adaptive response of polyprenols in Archaea. (**A**), Position and impacts of polyprenols on membrane physicochemical properties. (**B**), Structures and distribution of polyprenols within the Archaea domain. The tree topology has been adapted from [34]. In polyprenol structure, *m* refers to the number of cis isoprene units whereas *n* correspond to the trans units. The sum of m, n and alpha and omega units correspond to the total number of carbons, indicated in colors. Unsaturation degrees are indicated below the polar head group. (**C**), Pirateplot of the optimal growth conditions of the organisms in which the different lengths of polyprenols were detected. Colors indicate the chain lengths as in B. Abbreviations: EURY1, Euryarchaeota cluster I; PROTEO, Proteoarchaeota; EURY2, Euryarchaeota cluster II.

As critical lipid carriers for membrane protein glycosylation, polyprenols were looked for in the three domains of life and are somewhat well documented in Archaea. As mentioned above, Bacteria mostly produce polyprenols with 11 isoprene units, even though molecules with eight to 12 units have been reported [70,71]. Gram-negative bacteria synthesize only polyprenyl-alcohols, whereas phosphate derivatives dominate in gram-positive bacteria [68]. In contrast to Bacteria, Eukaryotes produce dolichols of a wider range of chain lengths. For instance, dolichols possess 18 to 21 isoprene units in mammals and dolichols with up to 40 units were detected in plants [72,73]. Although the proportions might vary according to cell types, phosphate derivatives remain the dominant form of polyprenols in Eukaryotes [68]. Nevertheless, dolichyl-alcohol may represent up to 90% of the dolichol derivatives’ pool, even though their function still remains unclear [74]. In contrast to Bacteria, in which the alpha-isoprene unit is unsaturated, and to Eukarya, in which the alpha-isoprene unit is the only saturated isoprene, dolichols with both saturated alpha- and omega-isoprene units were detected in every Archaea [75] (Figure 3B). Dolichyl-phosphates have been identified as the most physiologically relevant form of polyprenol derivatives in Archaea, and are present in all groups studied to date. Similarly to Bacteria, the most common polyprenols in Archaea consist of 11 isoprene units, but polyprenols with six to 14 units were detected in *Sulfolobus acidocaldarius* [76] and *Pyrococcus furiosus* [77] (Figure 3B). Apart from the alpha- and omega-isoprene units, the degree of unsaturation is highly variable, ranging from fully saturated to fully unsaturated molecules, even in a single archaeal species, such as *Sulfolobus acidocaldarius* [76]. Thus, although only polyprenyl-phosphates might be present in archaeal membranes, which may partake in protein glycosylation, a large variety of polyprenol side chain structures of yet-unidentified function has been revealed in Archaea (Figure 3B). Interestingly, dolichyl-alcohols were also detected in Archaea, but only in the methanogenic archaea Methanobacteriales and Methanomassillicoccales [78,79] while the rest of the methanogens, such as Methanococcales and Methanosarcinales, produce dolichyl-phosphates [80,81]. However, it should be considered that dolichyl-alcohols were only observed when using an acidification step during lipid extraction [78,79], which may have led to the hydrolysis of the polar head group and introduce an analytical bias [78,82]. Dolichyl-diphosphates were identified only in Crenarchaeota [83], whereas dolichyl-monophosphates were found in every other Archaea (Figure 3B) [84]. In addition, we show here that the euryarchaeal cluster I/proteoarchaea species tend to produce shorter polyprenols, i.e., around 10 isoprene units, than their counterparts of the euryarchaeal cluster II, i.e., around 12 units (Figure 3B). Similarly to Crenarchaeota, Eukaryotes also use long, dolichyl-diphosphates, which further supports the putative proteoarchaeotal ancestry of Eukaryotes [85]. Further studies of archaeal polyprenols, and especially within Crenarchaeota, would thus shed light on one of the currently most debated topics in evolutionary biology.

### 6.2. Biological Function of Polyprenols

Polyprenols are key components of the membrane protein glycosylation pathway and are thus required for the proper biosynthesis of critical cell structures, such as cell wall of various Bacteria [86] and eukaryotic spores [87]. In addition, polyprenols have been suggested to stabilize protein domains and complexes [88,89] and act as antioxidants that scavenge free radical oxygen species to protect surrounding lipids from peroxidation [90]. Altogether, these results indicate that polyprenol derivatives may have direct and indirect roles in tolerance to environmental conditions.

### 6.3. Insertion of Polyprenols in the Membrane

The less polar residues, i.e., polyprenyl-alcohols, tend to form aggregates that are horizontally buried in the membrane, whereas the more polar residues, i.e., polyprenyl-phosphates, are dispersed and vertically anchored with their polar head placed in the polar region of the membrane [91,92,93,94] (Figure 3A). Despite variable length, all studies demonstrate that the structural characteristics of polyprenols are strikingly analogous. For example, C55 and C95 polyprenols both compress their long hydrophobic tail into a similar chair-like conformation, such that they both only penetrate a single membrane leaflet [69,89,94,95]. Consequently, even though polyprenyl-phosphates harbor various side chain length, they may similarly impact membrane properties in the three domains of life.

### 6.4. Polyprenols as Putative Membrane Regulators in Archaea

Insertion of polyprenols into model membranes demonstrated that they might form aggregates, or domains, that could exert critical structural, functional and metabolic consequences on lipid bilayers [96]. In contrast to cholesterol, polar polyprenols were suggested to decrease the temperature of the lamellar-to-hexagonal phase transition, probably by specifically associating with phosphatidylethanolamine lipids [97]. For instance, polyprenols promote non-bilayer phase formation [92,97,98], and thus the formation and the fusion of membrane vesicles [99,100]. However, the effects of polyprenols on lipid membrane parameters appear to be dependent on the length of their polyisoprenoid chains [97]. Polyprenols with longer chain, such as C120 and C160 homologs, increase the thickness of the membrane hydrophobic core and lipid motional freedom [91], enhance membrane fluidity [98,101,102], and drastically increase ion [103,104] and water permeabilities [97,98,105]. In contrast, shorter chain polyprenols, such as C55 and C95, promote hydrophobic interactions with the lipid acyl chains [95], and thus reduce water permeability, especially in polyprenyl-phosphate based membranes [99]. In plants, these medium-chain polyprenols reduce lipid acyl chain motion, and thus membrane fluidity, in the protein-dense thylakoid membranes [106,107]. Although polar headgroups appear to greatly impact the membrane location of polyprenols, no study has been performed to estimate their impact on membrane parameters. Last, it is important to point out that all studies were performed in conditions (1% to 20% of polyprenols) far from those found in natural biological systems (less than 0.1%) [68], which implies that all effects of polyprenols on membrane regulation may not have been identified yet. To date, there is little evidence of a link between polyprenols and a response to environmental stressors. However, plants accumulate polyprenols in response to hypersaline stress [108] and a polyprenol kinase mutant of *Streptococcus mutans* exhibited a higher sensitivity to acidic conditions [109], suggesting that polyprenyl-phosphates may be involved in stress response. In Archaea, different polyprenol side chain structures were correlated with optimal growth conditions (Figure 3C). Archaea thriving at higher temperatures tend to produce polyprenols with shorter side chains, which sounds consistent with the tightening impact of short chain polyprenols on membranes. Halophilic environments are extremely unfavorable to bioenergetics [110]. However, halophilic archaea produce long polyprenols, with up to 12 isoprene units [111], which reinforce membrane impermeability to ions and thus allow to create gradients [110]. Polyprenol chain length did not seem to correlate with the optimal pH. However, hyperthermophilic archaea, such as *Pyrococcus furiosus* (T_opt_ ≈ 100 °C) and *Sulfolobus acidocaldarius* (T_opt_ ≈ 80 °C), produce highly saturated dolichyl-phosphates [76,77] (Figure 3B), which suggests that the number of unsaturations in polyprenyl-alcohols is also part of the adaptive response to extreme conditions, as demonstrated for membrane lipids [112].

## 7. Quinones

### 7.1. Distribution in Archaea

**Figure 4:**
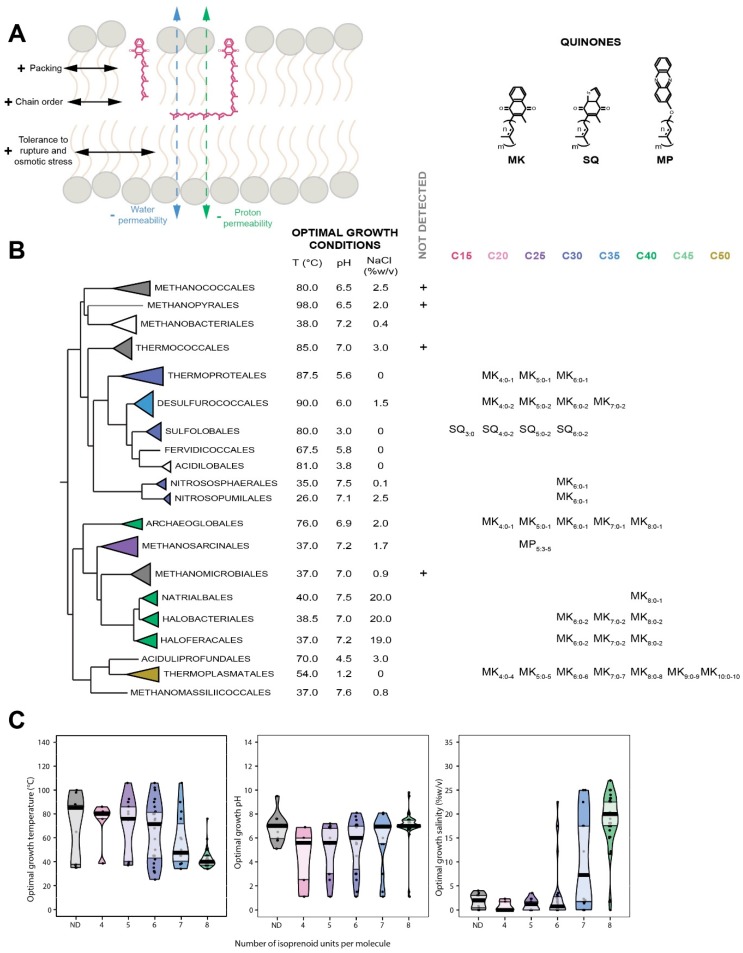
Physicochemistry, distribution and adaptive response of quinones in Archaea. (**A**), Position and impacts of quinones on membrane physicochemical properties. (**B**), Structures and distribution of quinones within the Archaea domain. The tree topology has been adapted from [34]. The different chain lengths are represented and the polar heads and insaturation degrees are indicated. (**C**), Pirateplot of the optimal growth conditions of the organisms in which the different side chain lengths of quinones were detected. Colors indicate the quinone apolar chain lengths as in B. Abbreviations: MK, menaquinone; SQ, sulfolobusquinone; MP, methanophenazine. EURY1, Euryarchaeota cluster I; PROTEO, Proteoarchaeota; EURY2, Euryarchaeota cluster II.

Quinones are a diverse group of membrane-bound amphiphilic isoprenoid derivatives ensuring electron and proton transfers in the respiratory chains of organisms throughout the entire tree of life. Due to their key position in the central metabolism of the cell, quinones are prevalent in all three domains of life, although their polar headgroups and side chain lengths vary. The cycles of the polar headgroups are used to classify quinones into benzoquinones, such as ubiquinones (Ub) and plastoquinones [113,114], naphtoquinones, such as phylloquinones and menaquinones (MK) [113,115] and sulfolobusquinones (SQ) [115]. Methanophenazines (MP) [116], which are analogous to quinones both in structure and function, are also included in this classification. Animals and plants synthesize long-chain ubiquinones, mainly Ub_10_, whereas fungi synthesize Ub_6_ to Ub_10_ [114]. Some Eukarya also synthesize phylloquinones or plastoquinones found in chloroplastic membranes [117]. Bacteria synthesize Ub and MK with side chains ranging from six to 10 prenyl units [118]. In contrast, most archaea produce short-chain MK with four to eight prenyl units, although two groups of archaea synthesize specific quinones: SQ with side chain of three to six units in Sulfolobales [119] and MP with five prenyl units in Methanosarcinales [120] (Figure 4B). Some archaea may synthesize long menaquinones such as MK_9_ and MK_10_ [121]. The nomenclature of quinones used in the current review (Q_m:n_) describes the polar headgroup (Q), the size of the isoprenoid side chain (m) and its number of unsaturations (n).

### 7.2. Biological Function of Quinones

The main function of quinones is to ensure the transfer of electrons and protons in the respiratory chains in the plasma membrane. The quinone polar headgroups composed of cyclic groups with distinct redox potentials constitute their critical biological moiety. On the other hand, the quinone apolar region is composed of polyisoprenoid side chains varying in size and saturation degree and is supposed to serve as an anchor in the membrane much like for polyprenols.

### 7.3. Insertion of Quinones in the Membrane

Despite a great deal of effort, the exact location of the quinone in the membrane remains uncertain. It seems clear that it is independent of the nature of the polar headgroup but is strongly impacted by the quinone side chain length [122,123,124,125]. Short-chain quinones, e.g., with an isoprenyl side chain no longer than the lipid acyl chain, lie parallel to the lipids while the quinones with longer chains, and especially Ub_10_, are progressively translocated within the midplane of the bilayer [126], regardless of the polar headgroup [125]. Most studies locate the polar headgroups of quinones like Ub_1_, Ub_2_, Ub_6_ or Ub_10_ into the bilayers with the quinone ring in the lipid polar head region, close to the glycerol moiety, thus quite distant from the lipid water interface [127,128,129]. Such position would explain quinone stability and lateral motility within a bilayer, but would require an energetically less favorable shift of the polar head from the inner to the outer membrane side (“flip-flop”) to allow the transfer of protons and electrons. In contrast, other studies locate Ub_10_ within the midplane of the bilayer [130], which would help enhance lateral diffusion but would be highly unstable, due to the insertion of a polar headgroup in the extremely hydrophobic core, and non-functional, as protons and electrons are supposed to transit from one membrane side to the other. Different simulations established that the location of the quinone may depend on its initial position, in the bilayer or in the midplane, and on the bilayer phospholipid composition. However, none could reproduce the fast shifts detected in mitochondrial bilayers [131,132]. Altogether, these results suggest that the location near the polar head region might actually be the most physiologically relevant quinone position, with their isoprenoid chains parallel to the lipids chains. The quinones whose polyprenyl chain lengths exceed that of the lipid acyl chains may lie in part in the midplane of the bilayer, similarly to long chain polyprenols [123].

### 7.4. Quinones as Putative Membrane Regulators in Archaea

**Figure 5:**
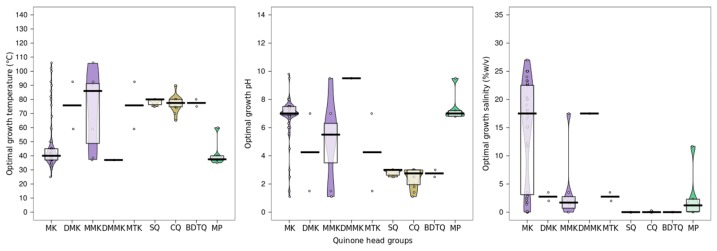
Correlation between optimal growth conditions and quinone head group. Menaquinone based quinones are depicted in purple (MK, DMK, MMK, DMMK, MTK), Sulfolobusquinone based quinones in yellow (SQ, CQ, BDTQ) and Methanophenazine (MP) in green. See the phylogenetic tree for head group structure. Abbreviations: MK, Menaquinones; DMK, Demethylmenaquinones; MMK, Monomethylmenaquinones; DMMK, Dimethylmenaquinones; MTK, Methionaquinones; SQ, Sulfolobusquinones; CQ, Caldariellaquinones; BDTQ, Benzodithiophenoquinones.

Apart from their main biological function, quinones have been suggested to act as membrane stabilizers and modulate membrane mechanical strength and permeability [133]. Long-chain ubiquinones increase packing and lipid order, thus limiting proton and sodium leakages and the release of hydrophobic components [126,134], and enhance the resistance to rupture and detergents [134,135] (Figure 4A). In contrast, quinones with short chain length, i.e., that do not exceed the lipid acyl chain, such as Ub_2_ and Ub_4_, drastically decrease melting temperatures [124]. Some archaea adapt their quinone content to correspond to the redox potential of their environment. For instance, *Thermoplasma acidophilum* produces a 1/1/1 ratio of Methionaquinones, Monomethylmenaquinones (MMK) and MK but almost exclusively MMK under aerobic and anaerobic conditions, respectively [136]. Similarly, the quinone composition is correlated to oxygen content and carbon source in *Sulfolobus solfataricus* [137] and *Acidianus ambivalens* [138]. However, despite growing under very different environmental conditions, most archaea possess identical quinone polar headgroups, suggesting that the polar moiety of archaeal quinone reflect the organism metabolic type rather than partake in membrane adaptation. Several line of evidence data demonstrate that quinone tails may participate in archaeal membrane adaptation, as demonstrated in Bacteria such as *Escherichia coli* [139] and *Listeria monocytogenes* [140] for the tolerance to osmotic shock or growth at low temperature. Indeed, various chain lengths, e.g., from 15 to 50 carbons, and saturation degrees, e.g., from fully saturated to one unsaturation per prenyl unit, were described in archaeal quinones, suggesting that the polyprenyl tails may support adaptive functions. If no specific study has tried to correlate the polyprenyl chain length and growth conditions in Archaea, we show here a clear correlation between polyprenyl chain length and optimal growth conditions in Archaea (Figure 4C). Interestingly, all environmental parameters, i.e., temperature, pH and salinity, seem to affect the size of quinone tails in Archaea, whereas no clear trend could be drawn for their polar heads (Figure 5). Under high temperature, Archaea tend to produce quinones with shorter side chains, which would be consistent with the proposition that the long isoprenoid chains, which are partially inserted in the midplane, tend to destabilize the bilayer, while the short chains, which only reside within the hydrophobic core of the leaflet, tend to improve membrane packing and lipid chain order. Similarly, the higher the salinity the longer the side chains, consistent with the suggested reduction of water, ion and proton permeabilities, all required in the deleterious bioenergetic landscape imposed by high salt conditions, associated with the long side chains which populate the midplane of the membrane bilayer [110].

## 8. Apolar Polyisoprenoids

Apolar polyisoprenoids, composed of four to eight isoprene subunits, are a vast and essential group of naturally occurring hydrocarbon compounds. As precursors of most terpenoids, apolar polyisoprenoids are present in all three domains of life. Squalene, formed by six terpenes subunits, is the precursor of steroland hopanoid derivatives [141,142,143], while lycopene, composed of eight terpenes subunits, is the precursor of carotenoids [144]. Although Archaea may lack some or all of these final products, apolar polyisoprenoids are broadly distributed in this domain [145], among which squalene is the most frequently found compound.

### 8.1. Distribution in Archaea

**Figure 6:**
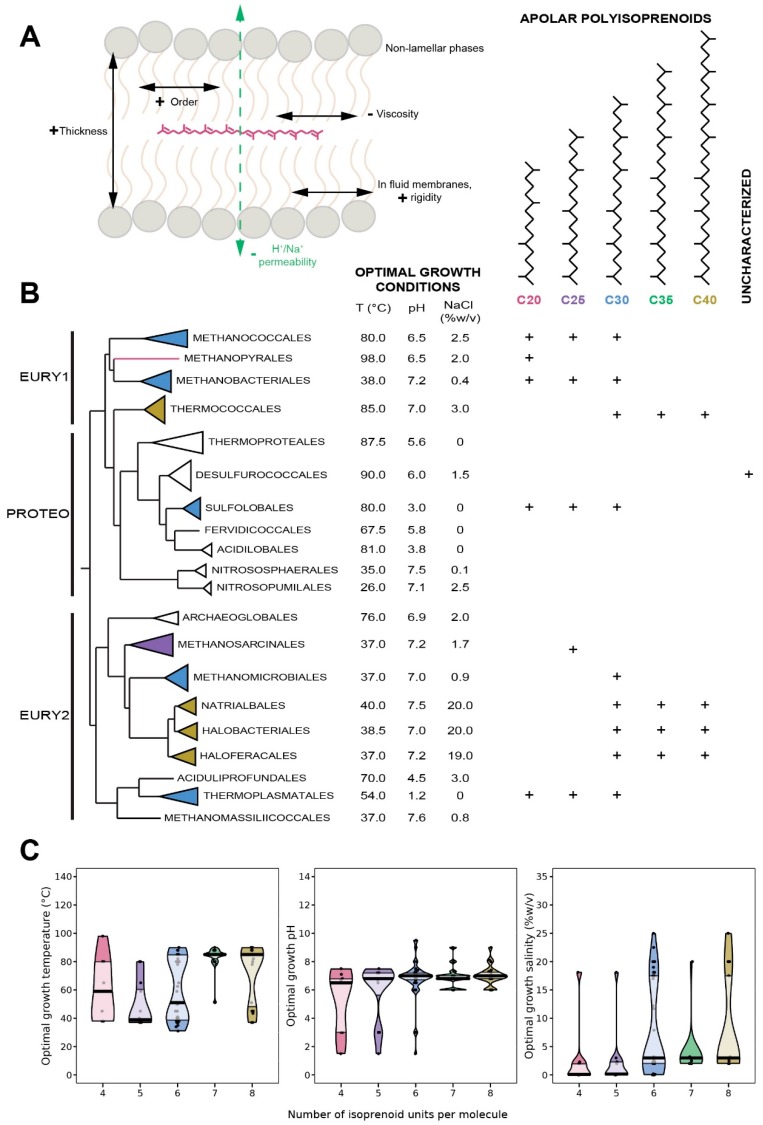
Physicochemistry, distribution and adaptive response of apolar polyisoprenoids in Archaea. (**A**), Position and impacts of apolar polyisoprenoids on membrane physicochemical properties. (**B**), Structures and distribution of apolar polyisoprenoids within the Archaea domain. The tree topology has been adapted from [34]. (**C**), Pirateplot of the optimal growth conditions of the organisms in which the different lengths of apolar polyisoprenoids were detected. Colors indicate the chain lengths as in B. Abbreviations: EURY1, Euryarchaeota cluster I; PROTEO, Proteoarchaeota; EURY2, Euryarchaeota cluster II.

As terpenoids precursors, apolar polyisoprenoids are broadly present in Eukarya, from shark (squalene) to tomatoes (lycopene) [146,147]. They are highly prevalent in acidophilic and alkaliphilic bacteria, which contain up to 40 mol% of polyisoprenoids, within which 10–12 mol% are of the squalene series [148,149]. Apolar polyisoprenoids with 4 to 8 units were identified in almost all the archaeal species tested, resulting in a broad distribution in the Archaea domain (Figure 6B). Archaea can be divided into two classes according to the length of their polyisoprenoids: (1) species that synthesizes short polyisoprenoids, with four to six isoprene units, such as Methanococcales and Sulfolobales, and (2) those that synthesize long polyisoprenoids, with six to eight isoprene units, e.g., Haloferacales and Thermococcales (Figure 6B). Although the proportions of apolar polyisoprenoids in archaeal membranes remain mostly unknown, it has been demonstrated that the linear isoprenoids of the lycopene series represent 1–2% of total lipids in *Thermococcus barophilus* [150] and 0.4% in *Thermococcus hydrothermalis* [151], two hyperthermophilic and piezophilic archaea. In addition, the degree of unsaturation of polyisoprenoids in Archaea remains poorly characterized.

### 8.2. Biological Function of Apolar Polyisoprenoids

Besides being synthetic intermediates for essential biomolecules, squalene has been studied as a possible antioxidant. Early studies have shown that squalene is an efficient singlet oxygen scavenging agent [152] and effectively protects lipid from peroxidation [153], but this antioxidant ability was recently challenged [154]. The structural similarity between apolar polyisoprenoids and the phytanyl chain constitutive of archaeal lipids [155,156] could lead to the belief that apolar polyisoprenoids are only intermediates in bipolar lipid biosynthesis or the products of polar lipid metabolism. However, both types of molecules derive from two different synthesis pathways: whereas the two phytanyl (C20) chains of bipolar archaeal lipids are linked through a 1-1 condensation, the central isoprene units of apolar polyisoprenoids and their derivatives result from the condensation of two polyprenyl-diphosphates through a 4-4 bound (Figure 1). Nonetheless, several studies suggest that apolar polyisoprenoids might be membrane regulators [150,157,158].

### 8.3. Insertion of Apolar Polyisoprenoids in the Membrane

Only a single study reports the localization of apolar polyisoprenoids in archaeal membranes. In membranes reconstructed from lipids of the archaeon *Halobacterium salinarum*, squalene was shown to prevent pyrelene, a fluorescent probe, to populate the midplane of the membrane bilayer, while reducing the local viscosity around the probe [159], which suggested that squalene was inserted in the hydrophobic core of the lipid bilayer perpendicularly to the membrane plane. However, neutron diffraction using squalane, the saturated form of squalene, and the eukaryal/bacterial phospholipid1,2-dioleoyl-sn-glycero-3-phosphocholine, demonstrated that squalane lies in the center of the lipid bilayer, parallel to the plane and partially extending into the lipid fatty acyl chains [158]. Our unpublished data confirm these later results in membranes reconstructed from different archaeal lipids, which allows us to generalize this localization for all bilayer-forming archaeal lipids (Salvador Castell/Oger unpublished results). In contrast to squalane, which is a flat molecule, squalene is more physically constrained due to the presence of unsaturations [160] and has several kinks, which indicates that it should remain more easily in the bilayer midplane and not insert within the hydrophobic core of the bilayer leaflets. The impact of the insertion of apolar polyisoprenoids on membrane parameters is yet to be reported for archaeal lipids, but several trends can be extrapolated from results with bacterial-like membranes (Figure 6A). First, the effects have been shown to vary with the lipid composition of the host membrane. Squalene increases rigidity of moderately fluid membranes, but has a rather softening effect on already rigid membranes [161]. In this context, in which squalene is in competition with other membrane regulators, such as cholesterol, membrane rigidity is driven by the squalene to sterol ratio [161]. In addition, apolar polyisoprenoids, such as squalane, squalene or lycopene, facilitate the formation of non-lamellar phases. For example, they induce the reduction of the lamellar-to-hexagonal phase transition temperature in bacterial-like [162,163], or archaeal-like membranes, indicating a stabilization of the non-lamellar phase. This signifies that apolar polyisoprenoids induce a higher negative curvature in the lipid bilayer, which could explain the higher aggregation and fusion of liposomes observed in the presence of lycopene [57]. In addition, chemical models predict that apolar polyisoprenoids populating the membrane midplane would decrease water, proton and sodium permeability. Thus, apolar polyisoprenoids could be essential to generate gradients to gain energy in more extreme environments, where these gradients may be harder to achieve [157]. This was partially confirmed with squalene in soybean membranes, demonstrating an altered proton pump [164], and with lycopene in bacterial-like liposomes showing increase water impermeability [139].

### 8.4. Apolar Polyisoprenoids as Putative Membrane Regulators in Archaea

There is a large body of evidence supporting a possible role of apolar polyisoprenoids as membrane regulators. To begin with, studies about lycopene contents of *Haloferax volcanii* and *Zymomonas mobilis* show that apolar polyisoprenoid levels are growth phase dependent, with increased synthesis in stationary phase [165,166]. The high energy-cost of lycopene synthesis, i.e., 24 ATP and 12 NADH per molecule, suggests that the presence of squalene-type isoprenoids must be essential for cell viability in the stationary phase. In addition, archaea adapt the degree of unsaturation of their apolar polyisoprenoids in response to environmental conditions. For instance, the level of unsaturation varies as a function of H_2_ availibity in cells grown in serum bottles vs. in fermenters [167]. In the polyextremophile *Thermococcus barophilus*, the degree of unsaturation of the pool of polyisoprenoids, from C_30_ to C_40_, is regulated in response to variations of temperature and hydrostatic pressure, which is part of the homeoviscous response of the membrane in this species along with variations in membrane polar lipids [150]. The novel membrane architecture proposed for *T. barophilus* suggests that apolar polyisoprenoids may be inserted in parallel to the membrane plane in the midplane of the archaeal bilayer. Thus, if validated, this architecture implies the existence of membrane domains of different compositions and properties, opening the possibility that the archaeal membrane can be spatially organized and functionalized (Figure 6C). In addition, the presence of the polyisoprenoids is supposed to compensate for the lack of monolayer-forming lipids in these species. Supporting this model, the length of apolar polyisoprenoids is positively correlated with (1) temperature, mesophilic archaea producing four to five isoprene long-hydrocarbons whereas hyperthermophilic microorganisms harbor six to eight isoprene units (Figure 6C); (2) pH, acidophiles synthesize shorter isoprenoid chains, i.e., C_20_ to C_30_, than alkaliphiles, i.e., C_30_ to C_40_ isoprenoids, and (3) salinity, halophiles present up to 40% of C_30_ to C_40_ apolar polyisoprenoids, longer than those of species thriving in low salinities [168]. Apolar polyisoprenoids may act by reducing the diffusion of ions and water across membranes, similarly to ring-based sterols [3,17,169]. As the proportion of tetraether, monolayer forming archaeal lipid is also correlated with temperature and pH, the long-chain polyisoprenoids are correlated with archaea harboring bilayers, in which the presence of polyisoprenoids would reinforce membrane resistance to stress. Altogether, our results suggest that apolar polyisoprenoids are essential membrane lipids of Archaea, allowing them to withstand a large range of stressful conditions, and behave as true membrane regulators.

## 9. Conclusions

**Figure 7:**
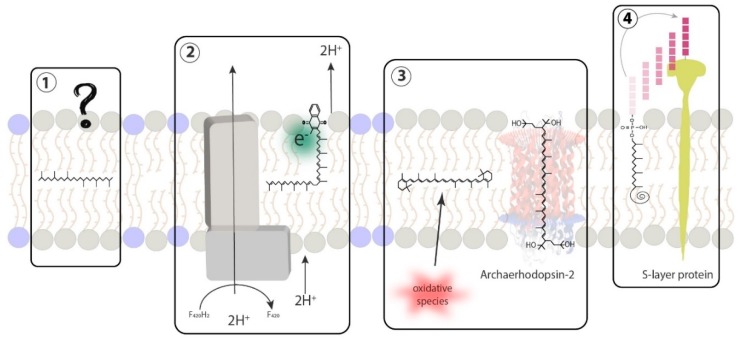
Schematic representation of major terpenoids found in Archaea and their respective biological functions. Blue and grey phospholipids represent archaeal tetraether and diether lipids, respectively. (1) squalane-type polyisoprenoids are widespread polyterpenes with yet-uncharacterized membrane function, (2) quinones are critical membrane-bound electron and proton carriers in energy transduction of various organisms, (3) carotenoids are well-characterized lipid-soluble antioxidants that may associate with transmembrane proteins and, (4) polyprenols are membrane-bound sugar carriers that are essential for transmembrane protein glycosylation.

Polyterpenes are a vast group of hydrocarbon compounds found in every known organism. Although they were initially described as having only physiological roles, such as pigments, hormones, protein regulators or energy transduction molecules (Figure 7), some of the most well-known polyterpenes, such as sterols and hopanoids, have been demonstrated to act as regulators of membrane properties.

Since Archaea are able to synthesize neither sterols nor hopanoids, the known membrane regulators, we looked for other polyterpenes that might support such membrane adaptive functions in Archaea. All four families of polyterpenes are synthesized by at least some species of Archaea. Although the data is quite scarce, it shows that all four type of molecules have the potential to act as membrane regulators in Archaea, and that they may be involved in the response to different stresses or in different branches of the Archaea. Of the four families, only quinones are not produced by all Archaea, and thus might not be the best surrogate of sterols or hopanoids. However, there is convincing evidence that the length of their polyisoprenoid tail influences membrane properties and that they are produced in specific compositions as a function of growth conditions. The carotenoids are very similar to quinones in their phylogenetic repartition, which seem restricted to certain branches of the Archaea, and their capability to impact on membrane properties. However, carotenoids have not been searched to the same extent as quinones, and it is thus possible that they might be more common than anticipated today. Their demonstrated properties in bacterial membranes are very similar to classical membrane regulators, such as reducing proton and water permeabilities. Thus, we anticipate a similar function in Archaea which synthesizes them, with a possible connection with the response to salinity and high pH stresses. Polyprenols, which constitute the third family, are also expected to lie vertically in the membrane with their long polyisoprenoid chains condensed into a chair-like conformation within the hydrophobic core of the membrane. Such conformation would affect the properties of the lipid bilayer, while being adapted as a function of different stressors, such as temperature or salinity. Apolar polyisoprenoids may be the best surrogates of sterols and hopanoids in Archaea, since they have already been demonstrated to enhance membrane properties to maintain membrane functionality in response to stress. However, further evidence will be required to demonstrate their presence and impact on membrane in all Archaea. Regardless of the family, the putative regulation effects of polyterpenes on membrane properties seem correlated with the length of their polyisoprenoid chains. As a general rule, archaea synthesizing monolayer-forming lipids tend to accumulate polyterpenes with shorter side chains, when the species forming bilayer membranes tend to accumulate polyterpenes with longer side chains. This is consistent with the model of Cario and colleagues who suggested that the polyisoprenoid chains may play a role equivalent to that of the monolayer-forming lipids, e.g., increased rigidity and impermeability to ions and water, shift in resistance to stress, in species incapable of synthesizing bipolar lipids [150].

**Figure 8:**
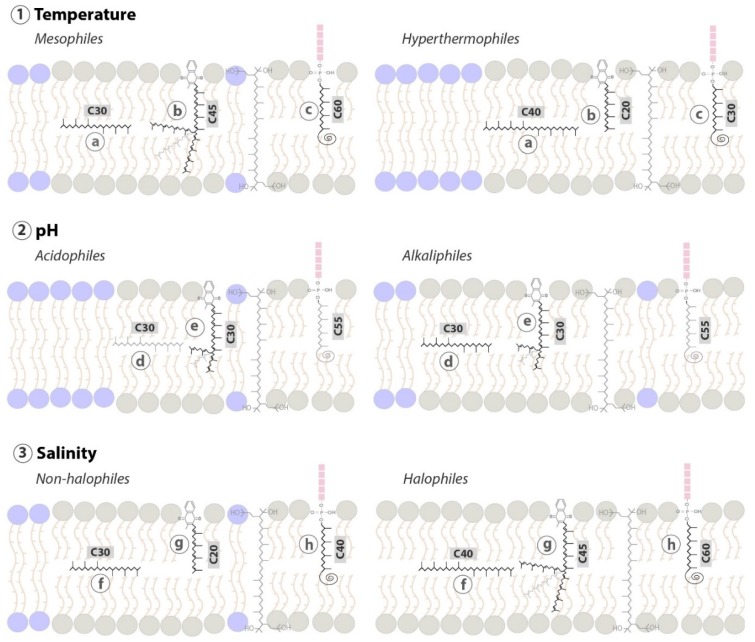
Schematic representation of putative membrane properties modulation by polyterpenes in response to different environmental conditions in Archaea. (**1**) Adaptations to temperature: (**a**) longer apolar polyisoprenoids are present in hyperthermophiles. (**b**) and (**c**) mesophiles present menaquinones and polyprenols with higher acyl chains than hyperthermophiles. (**2**) Adaptations to pH: (**d**) alkaliphiles possess higher quantities of apolar polyisoprenoids molecules. (**e**) Both, acidophiles and alkaliphiles own menaquinones with medium-length acyl chains. (**3**) Adaptations to salinity: (**f**) extreme halophiles present longer apolar polyisoprenoids lipids. (**g**) and (**h**) menaquinones and polyprenols with long acyl chain length are present in extremely halophilic archaea. Grey and blue molecules represent archaeal diether and tetraether lipids, respectively.

In Archaea, it is highly probable that more than one type of membrane regulator exists, much like in Eukarya or Bacteria, since the trend of accumulation of the different polyterpenes may be negatively correlated. For example, hyperthermophiles accumulate longer polyisoprenoids but shorter polyprenols and quinones than mesophiles, and acidophiles and alkaliphiles accommodate higher amounts of medium-length quinones, with alkaliphilic archaea possessing particularly large quantities of polyisoprenoids (Figure 8). More importantly, the presence of such lipid membrane regulators implies the existence of membrane domains, opening the possibility to the existence of functionalized regions in archaeal membranes.

## Abbreviations

IPP	Isopentenyl pyrophosphate
DMAP	Dimethylallyl diphosphate
T_m_	Lipid phase transition temperature
IDR	Isopentenyldehydrorhodopsin
BR	Bacterioruberin
BC	β-carotene
HEO	3-hydroechinenone
CTX	Canthaxanthin
AX	Astaxanthin
ZX	Zeaxanthin
EURY1	Euryarchaeota cluster I
PROTEO	Proteoarchaeota
EURY2	Euryarchaeota cluster II
T_opt_	Optimal growth temperature
Ub_x_	Ubiquinones and its number of isoprenoid units (x)
MK	Menaquinones
MMK	Monomethylmenaquinones
MTK	Methionaquinones
DMK	Demethylmenaquinones
DMMK	Dimethylmenaquinones
SQ	Sulfolobusquinones
CQ	Caldariellaquinones
BDTQ	Benzodithiophenoquinones
MP	Methanophenazines
Q_m:n_	Polar head group from quinone (Q), size of the isoprenoid side chain (m) and its number of unsaturations (n)
ATP	Adenosine triphosphate
NADH	Nicotinamide adenine dinucleotide
